# Seroepidemiology for *Orthorubulavirus suis* in Mexican Pigs by Development of an Indirect ELISA Based on a Recombinant NP Protein

**DOI:** 10.3390/pathogens13121135

**Published:** 2024-12-22

**Authors:** Rocío Lara-Romero, José Luis Cerriteño-Sánchez, María Azucena Castañeda-Montes, Humberto Ramírez-Mendoza, Julieta Sandra Cuevas-Romero

**Affiliations:** 1 Estancias Posdoctorales por México, Consejo Nacional de Humanidades, Ciencias y Tecnologías, Ciudad de México 03940, Mexico; lara.romero.mx@gmail.com; 2 Departamento de Microbiología e Inmunología, Facultad de Medicina, Veterinaria y Zootecnia, Universidad Nacional Autónoma de México, Ciudad de México 04510, Mexico; 3 Centro Nacional de Investigación Disciplinaria en Salud Animal e Inocuidad, Instituto Nacional de Investigaciones Forestales, Agrícolas y Pecuarias, Ciudad de México 05110, Mexico; cerriteno.jose@inifap.gob.mx; 4 Unidad Académica de Capulhuac, Universidad Tecnológica del Valle de Toluca (UTVT), Estado de México 52700, Mexico; azucena.castaneda.montes@gmail.com

**Keywords:** *Orthorubulavirus suis*, ELISA, recombinant nucleoprotein

## Abstract

*Orthorubulavirus suis* (LPMV) is the etiologic agent of blue eye disease (BED), which affects pigs of all ages, and it has been endemic in central Mexico since the 1980s. To date, no disease control program has been established. Therefore, there is a need for a serological diagnostic method with high sensitivity and specificity. In this study, the recombinant protein NP of LPMV was produced in the *E. coli* BL21 system and then purified using affinity chromatography. The purified protein was used to coat plates for an indirect ELISA assay (iELISA). To determine the sensitivity and specificity of the test, a 2 × 2 contingency table was constructed using positive and negative control sera. The specificity and sensitivity levels were 98.1% and 98.7%, respectively. According to our findings, 45% of serum samples (378/839) were positive, with seropositivity percentages in the analyzed states ranging from 72.5% to 6%. To confirm the presence of antibodies, the indirect immunofluorescence technique was applied to iELISA-positive serum samples. In this study, antibodies against the LPMV nucleoprotein were detected, indicating that the virus or defective particles may be circulating in Mexican pigs and highlighting the risk of LPMV spreading to disease-free areas.

## 1. Introduction

*Orthorubulavirus suis* is a paramyxovirus with an enveloped, negative-sense, single-stranded RNA genome, also known as La Piedad Michoacán Virus (LPMV), and is the causal agent of blue eye disease (BED) in pigs. Until 2022, according to its taxonomic classification, it was called *Porcine orthorubulavirus*. Before 2019, it was known as *Porcine rubulavirus* [1,2,3]. BED first occurred in 1980 on a pig farm located in La Piedad Michoacán, Mexico. In piglets younger than 21 days, BED causes a clinical picture of incoordination, muscle tremors, prostration, running movements, and corneal opacity. In pigs of all ages, 1–10% unilateral or bilateral corneal opacity (blue eye) may occur due to corneal edema and anterior uveitis [4,5]. In boars, lesions caused by BED result in inflammation of the head of the epididymis at 15 days post-infection (DPI) and reductions in sperm concentration and motility at 21 DPI; they are also associated with the formation of granulomas, and the development of orchitis and fibrosis, at 70–80 DPI. Even when the infection becomes chronic, degeneration of the seminiferous tubules may be exhibited, along with lymphocytic infiltration (30 DPI) and testicular atrophy [6]. In adult sows, BED infections result in lower conception rates due to higher incidences of returns to estrus, abortions, fetal deaths, stillbirths, and mummified fetuses [7]. In the Mexican state of Jalisco, atypical outbreaks of BED occurred from 2000 to 2003 at a total of 22 multi-site farms. During these outbreaks, 60% of 3–4-month-old pigs presented pneumonic signs, 20% of these animals presented signs of encephalitis, and mortality increased by 30%. This increase in LPMV neurovirulence may be associated with mutations observed in the LPMV HN gene in the PAC6-PAC9 variants [8]. In 2013, the pathogenicity and distribution of LPMV infection in the respiratory tract of pigs was first evidenced. Viral shedding was detected in nasal fluids up to 23 DPI, and high viral loads were detected in the tonsils, soft palate, and lymphoid nodules. The main lesions were in the lungs and corresponded to interstitial pneumonia and hyperplasia associated with lymphoid tissue [9]. LPMV is made up of 15,180 nucleotides, which act as a template for the synthesis of messenger RNA and the antiviral genome [10]. It is divided into six genes (3′-NP-P-M-F-HN-L-5′) that code for six structural proteins and three non-structural proteins. Of the structural proteins, three are associated with the nucleocapsid, namely the nucleoprotein (NP), the phosphoprotein (P), and the high-molecular-weight protein (L), and three are associated with the LPMV membrane, namely the matrix protein (M), hemagglutinin-neuraminidase (HN), and the fusion protein (F) [11]. The humoral response against LPMV begins with the production of antibodies from the first week post-infection. During the first four weeks, the titers increase to between 4 and 6 logarithmic units (base 2), and from the fifth week, they increase up to 8.5 logarithmic units. The antibodies generated are mainly directed against the HN, NP, and M proteins, although the specificity and immunodominance of the humoral response towards the HN protein of the virus have been reported [12]. BED has only been reported in Mexico, where it remains endemic in the central states of the country, a situation related to the high population density of pigs, the limited local application of biosecurity measures, and poor controls when introducing animals to farms [13]. Using the serological technique of hemagglutination inhibition, researchers have found antigenic differences between different strains of LPMV, which are due to a lack of heterologous specificity, probably resulting from changes in antigenic regions of the HN protein. On the other hand, seroprevalence rates of between 9% and 23.7% have been reported for four central states of Mexico [14]. LPMV has the capacity to establish persistent infections leading to chronic disease, with the risk that persistently infected animals release the virus spontaneously [15]. In our research group, we have succeeded in producing recombinant NP and M proteins, which are antigenically recognized by antibodies from pigs experimentally infected with LPMV from 5 days post-infection, and even during persistence periods [16]. In light of the above, the goal of our study was to develop an indirect ELISA test (iELISA) with high sensitivity and specificity, using the recombinant NP protein of LPMV as an antigen, which is both conserved and antigenic. Using this technique, pig sera from the central states of Mexico were evaluated to obtain new information on seropositivity in this region.

## 2. Materials and Methods

### 2.1. Production and Purification of Recombinant NP-LPMV Protein

**Figure 1:**
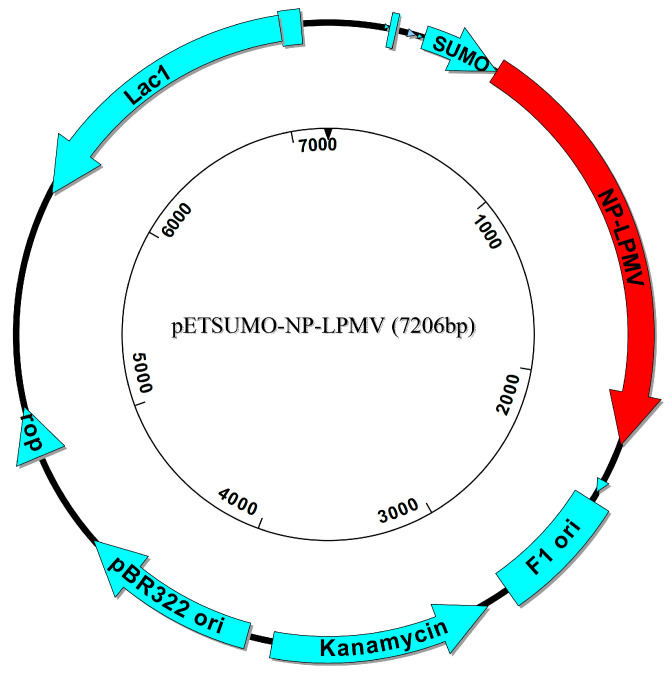
Map and features of pET SUMO-NP-LPMV vector construction. The red arrow indicates the cloned NP gene.

Recombinant protein NP-LPMV (*r*NP-LPMV) in Champion™ pET SUMO Expression System (Carlsbad, CA, USA, Thermo Fisher Scientific Inc.) (Figure 1) was expressed and produced according to previously established protocols [16]. Briefly, 100 mL of Luria–Bertani (LB) medium was inoculated with transformed *E. coli* BL21 starting at 0.1 optical density (OD) units at 600 nm; then, it was incubated at 37 °C and 250 rpm and induced when the culture reached 0.5 units at OD600 nm with 1.5 M IPTG (isopropyl-D-1-thiogalactopyranoside). Protein production was visualized using SDS-PAGE after staining with Coomassie Brilliant Blue G-250 (Bio-Rad, Hercules, CA, USA). The recombinant protein was confirmed by Western blotting using the 6X-His monoclonal antibody (Invitrogen, Rockford, IL, USA). The overproduction of recombinant proteins in *E. coli* was recovered from the inclusion bodies and purified according to Castañeda-Montes [17]. Briefly, the inclusion bodies were solubilized in N-Lauroylsarcosine 7% and Tris-HCl 50 mM, pH 8 buffer. Subsequently, the recombinant protein was purified using a Ni-NTA agarose column (5 mL) HisTrap^®^ Chelating High Performance (GE Healthcare, Chicago, IL, USA), and the pure rNP-LPMV was dialyzed against 300 volumes of Tris-HCl 5 mM, pH 7.5, with 8 kDa regenerated cellulose membrane. Finally, the presence of purified *r*NP-LPMV protein was confirmed using SDS-PAGE and Western blotting, and protein concentration was determined via the Bradford assay [18].

### 2.2. Standard Reference Serum

A set of sera from experimentally infected pigs were used as positive controls (*n* = 77), and another batch of sera from healthy pigs without a history of BED as negative controls (*n* = 104) to standardize the iELISA. All of the serum samples (*n* = 181) were obtained from previous work where pigs were handled at the facilities of the National Center for Disciplinary Research in Animal Health and Safety (CENID-SAI) of the National Institute of Forestry, Agriculture and Livestock Research (INIFAP), Mexico City, Mexico. All procedures were carried out in accordance with Mexican legislation (NOM-062-ZOO-1999; SAGARPA), based on the Guide for the Care and Use of Laboratory Animals, NRC [19]. Sera were tested by Western blot (WB) as a gold standard test following the reported methodology [20].

### 2.3. iELISA Test

iELISA testing was carried out to examine the reactivity of specific IgG antibodies to recombinant *r*NP-LPMV protein. Briefly, MaxiSorpTM 96-well microplates (Nunc Intl., Rochester, NY, USA) were coated with 100 μL of recombinant antigen at a concentration of 75 ng/mL in 0.05 M carbonate buffer (pH 9.5) and incubated overnight at 4 °C. The plates were blocked with 5% skimmed milk in PBS and then incubated for 1 h at 37 °C with serum samples diluted 1:200 in PBS-Tween 20 0.5%. After washing, the plates were incubated with horseradish peroxidase (HRP)-labeled anti-pig-IgG (Sigma, St Louis, MO USA), and the chromogenic reaction was developed using 3,3′,5,5′-tetramethylbenzidine TMB substrate (Sera-Care, Milford, MA, USA). Optical density (OD) was measured in a microplate reader at a wavelength of 450 nm [21].

### 2.4. Statistical Analysis

The evaluation of the ELISA tests, to determine sensitivity and specificity values, was carried out in comparison with the results of the reference WB test using a 2 × 2 contingency table (Table 1) [20]. Validation of the immunoassay was achieved using a concordance analysis with respect to the reference test. The level of agreement between the two tests was determined using kappa values, which measure the level of agreement between tests beyond chance. A kappa value of 0 indicates that there is no agreement between the tests evaluated; a kappa value greater than 0.8 indicates very good agreement [22]. Plots and maps were constructed using SigmaPlot version 12.5 (Systat Software Inc., San Jose, CA, USA.

Using the optimal conditions of the iELISA, a total of 839 serum samples were expressed as percentage positivity for diagnostic interpretation [21].

### 2.5. Seroepidemiological Analysis of Field Serum Samples

In order to determine the serological distribution of LPMV in central Mexico, 839 blood samples were taken to obtain sera. Samples were taken from the following states: Aguascalientes (*n* = 182), Guanajuato (*n* = 108), Jalisco (*n* = 254), State of Mexico (northern and southern region) (*n* = 51), Michoacán (*n* = 35), Morelos (*n* = 73), Querétaro (*n* = 86), and Veracruz (*n* = 50). Samples were obtained from pigs, in the range of 5 to 7 months of age, from public slaughterhouses, backyard production facilities, and medium- and high-tech farms between 2019 and 2024 (Table 2). The samples obtained from slaughterhouses came from farms with at least 1000 sows, semi-technical farms with at least 100, and backyard units with at least 10 sows. The serum samples were stored at −20 °C until they were used in the iELISA and immunofluorescence tests.

### 2.6. Indirect Immunofluorescence Test on Field Serum Samples

An indirect immunofluorescence assay with modifications was performed on ovine choroid plexus (SCP) cell cultures infected with LPMV [23,24]. Briefly, SCP cell monolayers in 96-well plates were inoculated with 300 TDICC50% of LPMV strain PAC 3. Plates were incubated at 37 °C in a 5% CO_2_ atmosphere for 48 h and subsequently fixed in 4% paraformaldehyde. Samples used for IF testing were positive and negative sera detected at each stage using the iELISA test with recombinant NP protein. Plates were then washed with distilled water. Serum samples diluted 1:40 in PBS were then placed onto them and allowed to incubate at 37 °C for 30 min. Another wash was then performed. The secondary antibody Fluorescein (FITC)-Conjugated AffiniPure Goat Anti-Swine IgG (H + L) (Jackson ImmunoResearch Inc., West Grove, PA, USA) was then placed on plates at a dilution of 1:320 and then incubated at 37 °C for 30 min. Serum samples were considered positive when cells exhibited specific intracytoplasmic fluorescence.

## 3. Results

### 3.1. Production and Purification of the rNP-LPMV

**Figure 2:**
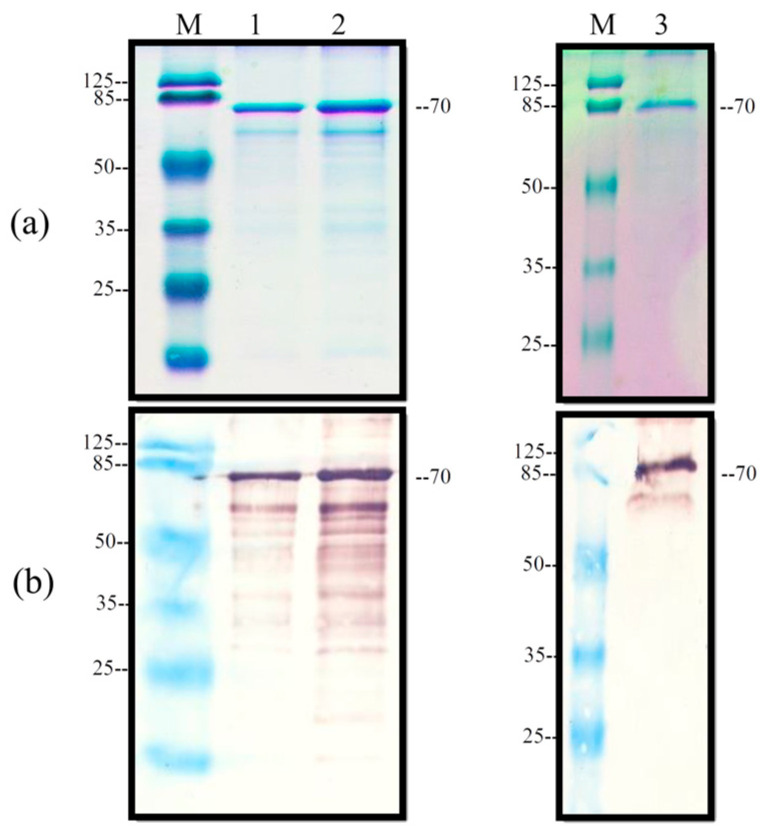
Overproduction of *r*NP-LPMV. (**a**) Coomassie staining and (**b**) Western blot with specific anti-his antibodies. (1) and (2) inclusion bodies from induced BL21-NP-LPMV; (3) *r*NP-LPMV purified by affinity chromatography.

The *r*NP-LPMV was expressed and produced in the pET SUMO vector using the *E. coli* BL21 system induced with IPTG. A predicted weight of 70 kDa was thereby achieved. When analyzing the fractions of the bacterial rupture, it was found that the insoluble fraction contained the largest amount of recombinant protein; this was due to the formation of inclusion bodies that were solubilized and that carried out the purification process by affinity chromatography. In the elution process, the purified *r*NP-LPMV was obtained at the expected weight and exhibited some degradations typical of the recombinant protein (Figure 2).

### 3.2. Standardization of the iELISA Test

**Figure 3:**
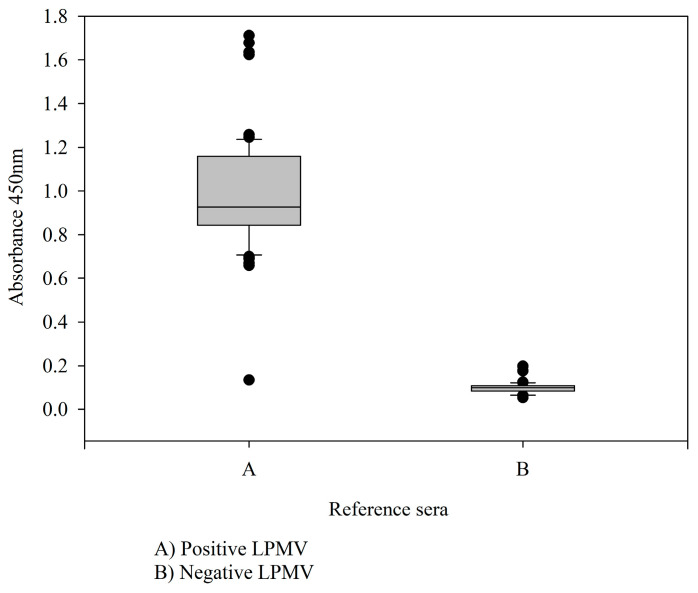
Distribution of OD450 mn of positive and negative reference sera in the *r*NP-LPMV iELISA test represented by box-and-whisker plot.

**Figure 4:**
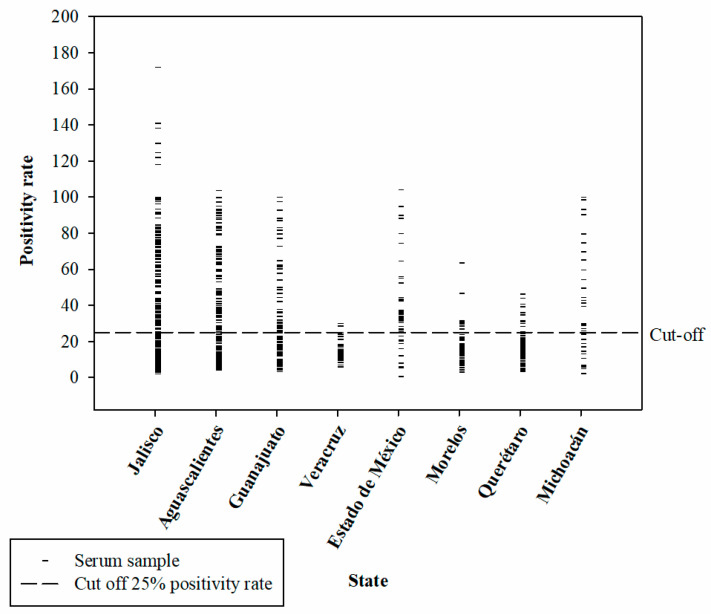
Distribution of the positivity rate of the serum samples from the eight states of Mexico used in the present study. The dotted line indicates the cut-off point of the test.

Through a series of dilutions, it was determined that the best concentration for the iELISA test was 75 ng of antigen per well (*r*NP-LPMV), and the optimal dilution of sample sera was 1:200. The secondary antibody was used at 1:17,500, with an incubation time of 1 h for each antibody at a temperature of 37 °C. After the substrate (TMB) was placed, a reading was taken after 10 min. All plates were blocked with a 5% skimmed milk solution. For validation of the iELISA test, the results obtained for the optical densities at 450 nm of the 181 reference serum samples were used (Figure 3), in order to establish the sensitivity and specificity values using the 2 × 2 contingency table (Table 3). The results of the iELISA showed a sensitivity of 98.7%, a specificity of 98.1%, and kappa index = 0.97. This assay can therefore detect infection at an early stage in pigs. The iELISA cut-off values, expressed as a percentage of positivity, were determined using a selected number of negative, positive, and weakly positive serum samples. The iELISA was standardized on large quantities of sera from field pigs from Mexico (Figure 4) and on sera from experimentally infected animals.

### 3.3. iELISA Analysis of Collected Serum Samples

**Figure 5:**
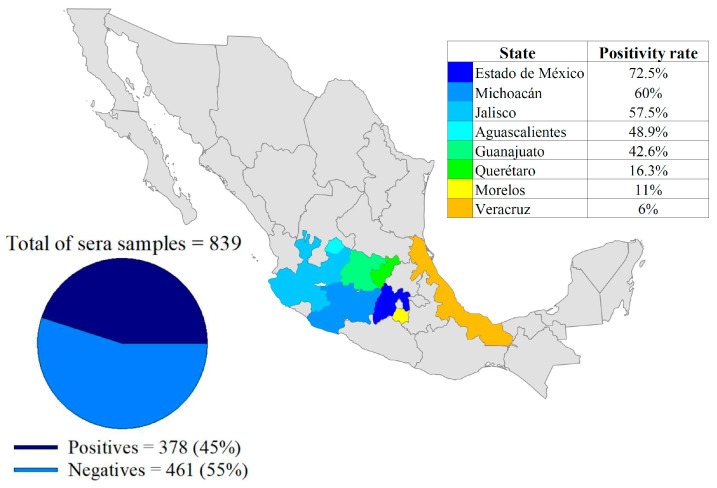
Map of Mexico showing the central states of study indicating the positivity rate of the serum samples.

Of the 839 serum samples collected from eight states in Mexico (Table S1), 45% (378) were positive. The state of Mexico had the highest positivity rate with 72.5% (37/51), followed by Michoacán with 60% (21/35), Jalisco with 57.5% (146/254), Aguascalientes with 48.9% (89/182), Guanajuato with 42.6% (46/108), Querétaro with 16.3% (14/86), Morelos with 11% (8/73), and, lastly, Veracruz with 6% (3/50) (Figure 5).

### 3.4. Identification of Antibodies Using Indirect Immunofluorescence

**Figure 6:**
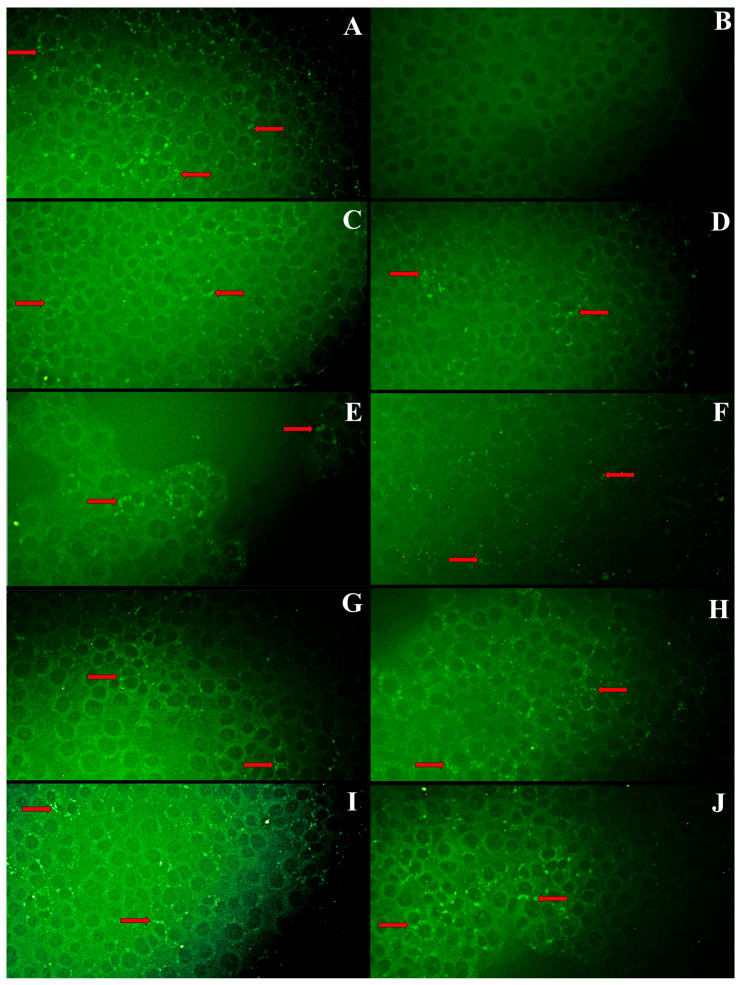
Indirect immunofluorescence in SCP cell culture observed at 20×. (**A**) Positive hyperimmune control serum. (**B**) Negative reference serum. (**C**) Positive sample from the State of Mexico. (**D**) Positive serum from Michoacán. (**E**) Positive serum from Jalisco. (**F**) Positive serum from Aguascalientes. (**G**) Positive serum from Guanajuato. (**H**) Positive serum from Querétaro. (**I**) Positive serum from Morelos. (**J**) Positive serum from Veracruz. Red arrows indicate some specific fluorescent foci.

The existence of LPMV infection in the SCP cell monolayer was evidenced by the presence of intracytoplasmic fluorescent granules due to specific antibody recognition in the positive control serum (Figure 6); however, the serum negative for antibodies against LPMV did not show specific fluorescence. In sera found to be positive using iELISA and selected on the basis of their status, we were able to recognize and evidence the presence of antibodies by observing multiple fluorescent granules.

## 4. Discussion

BED first appeared in pigs in the 1980s in the Mexican state of Michoacán and subsequently spread to the central states of Mexico, where the high population density of pigs and the lack of biosecurity measures have caused the disease to become endemic, resulting in the generation of viral strains capable of maintaining similar virulence characteristics, which continue to cause serious economic losses [25]. In 2023, Servicio de Información Agroalimentaria y Pesquera (SIAP) reported that the value of pig production in Mexico amounted to approximately USD 4017 million. Of this total, USD 1984 million (48.5%) was generated in the states evaluated in this work, highlighting the high level of economic risk posed by BED to this region [26]. Because there is no official standard or program for the control and eradication of BED, there is a lack of control due to the absence of a universal diagnostic method that can be used in areas free of the disease; therefore, the recombinant protein of LPMV, NP, has been proposed as an alternative for use in the development of diagnostic tools, due to its ability to detect specific antibodies from five days post-infection [16]. Because the NP of LPMV is a more conserved protein compared with HN, it was ideal for use as an antigen in the iELISA test conducted in the present work. Using recombinant nucleoprotein from the transmissible gastroenteritis virus of pigs, sensitivity and specificity values of 98.6% and 98%, respectively, were recorded, without cross-reactions with other viruses [27]. In another study, an iELISA using a recombinant NP protein was carried out for the detection of IgG against SARS-CoV2; in this work, high sensitivity and specificity were again obtained, without cross-reactions with specific antibodies against other pathogens [28]. Today, the serological technique most commonly used on an unofficial basis by laboratories is hemagglutination inhibition; specificity and sensitivity values of 100% and 89%, respectively, have been obtained using this method. However, to obtain such high values, it is necessary to pre-treat the sera to eliminate nonspecific components and inhibit the complement system [29]. This results in longer testing times compared with the iELISA assay with recombinant NP protein, which is performed within a period of 3 h, does not require pre-treatment of the serum samples, and delivers sensitivity and specificity values of 98.7% and 98.1%, respectively. Among other tests reported for the detection of LPMV, the blocking ELISA system is especially noteworthy. This method involves the use of monoclonal antibodies (mABs), and sensitivity and specificity values of 99% and 97% [30], respectively, have been obtained using this technique. However, costs increase when using mABs [31], which makes the blocking ELISA system expensive and unaffordable for pig producers. The strategies and methodologies currently used for the production of recombinant proteins involve costs that are competitive with products already offered on the market [32]. Our assay requires an amount of 7.5 μg to prepare a 96-well plate with antigen; this is very low cost because 20 plates can be obtained for diagnosis from a 100 mL culture according to the yields reported in the production of the *r*NP-LPMV [16]. Another iELISA test for the detection of LPMV was reported with a sensitivity and specificity of 100% due to the use of homologous positive sera using the recombinant protein HN as an antigen [33]. In the present study, the number of positive sera used was greater, and these sera were obtained from animals experimentally infected with field viruses, allowing the hypothesis that *r*NP-LPMV is a better antigen for diagnosis. In 2011, a hemagglutination inhibition test covering four central states of Mexico revealed seroprevalence of 23.7% to 9%; poor cross-antigenicity was also found when three LPMV isolates were compared, due to antigenic variations between the different circulating strains [14]. In our study, we obtained positivity rates ranging from 72.5% to 6% in the eight states we considered, with the state of Mexico having the highest number of LPMV-positive animals; however, it has seven geographic regions, of which only two were sampled (northern and southern regions), these data suggest deficiencies in biosecurity measures and lack of disease control. The next highest ranked states were Michoacán, Jalisco, and Guanajuato, all of which have a high population density of pigs, which makes it difficult to control the disease in swine. Despite the small number of farms and animals tested in Michoacán, the results suggest that seropositivity remains high, as it is the region of origin of LPMV. Production units in Aguascalientes are also affected, with 48.9% positivity, due to their proximity to large pig-producing states, endemic for BED. Querétaro, Morelos, and Veracruz were found to have the lowest positivity rates, likely due to their more technologically advanced production methods and higher levels of biosecurity. For each state considered in the present study, indirect immunofluorescence was used as a confirmatory test to demonstrate the presence of specific antibodies, because this is a reliable technique that is capable of detecting antibodies from eight days post-infection [34].

## 5. Conclusions

In this study, we used an iELISA test with high sensitivity and specificity that allowed us to obtain data on the presence of antibodies in the states of central Mexico. This presence was confirmed using indirect immunofluorescence. Our findings show data on the seropositivity of unvaccinated pigs in seven states, suggesting that the antibodies detected came from animals that have been in contact with LPMV because the samples come from unvaccinated pigs. Therefore, the development of the recombinant NP iELISA test will allow an accurate diagnosis capable of providing the necessary knowledge to improve BED control strategies as an effective detection alternative that health authorities can use in future campaigns to control and eradicate the disease in the country.

## Figures and Tables

**Table 1:** The 2 × 2 contingency table for determining sensitivity and specificity. A = number of true positives, B = number of false positives, C = number of false negatives, D = number of true negatives. Sensitivity (%) = [100] × [A/(A + C)], specificity (%) = [100] × [D/(B + D)].

	LPMV Positive	LPMV Negative	Total
Positive serum	A	B	A + B
Negative serum	C	D	C + D
Total	A + C	B + D	N

**Table 2:** Characteristics of serum samples.

State	Sera Quantity	Sera Origin	Number of Farms Sampled
Aguascalientes	182	Slaughterhouse	20
Guanajuato	108	Slaughterhouse	20
State of Mexico	51	Backyard production, semi-technical farm	8
Jalisco	254	Slaughterhouse	20
Morelos	73	Backyard production	5
Queretaro	86	Backyard production, technified farm	17
Michoacán	35	Technified farm	3
Veracruz	50	Slaughterhouse	8
Total	839		101

**Table 3:** The 2 × 2 contingency table for obtaining sensitivity and specificity.

	LPMV Positive	LPMV Negative	Total
Positive serum	76 (A)	2 (B)	78 (A + B)
Negative serum	1 (C)	102 (D)	103 (C + D)
Total	77 (A + C)	104 (B + D)	181 (N)

## Data Availability

No new data were created or analyzed in this study. Data sharing is not applicable to this article.

## Supplementary Materials

The following supporting information can be downloaded at: https://www.mdpi.com/article/10.3390/pathogens13121135/s1, Table S1: Data collection of iELISA assay from all serum samples obtained from pig farms.
